# A young girl with right ovarian torsion and left ovarian ectopy

**DOI:** 10.1186/s13052-020-0811-y

**Published:** 2020-04-23

**Authors:** Giuliana Morabito, Alessandro Daidone, Flora Murru, Marianna Iaquinto, Elena Faleschini, Egidio Barbi, Giorgio Cozzi

**Affiliations:** 1 Institute for Maternal and Child Health - IRCCS “Burlo Garofolo”, Trieste, Italy; 2 University of Trieste, Trieste, Italy

**Keywords:** Mayer-Rokitansky-Kuster-Hauser syndrome, Ovarian torsion, Kidney agenesis, Aplasia uterus, Emergency, müllerian structures, Pediatric, ectopic ovary

## Abstract

**Background:**

Mayer-Rokitansky-Küster-Hauser (MRKHS) syndrome refers to congenital hypoplasia/aplasia of the uterus, the cervix and the upper 2/3 of the vagina, in females with normal ovaries and fallopian tubes, secondary sexual characteristics and 46 XX karyotype. This condition originates from abnormal development of Müller’s paramesonephric ducts in the early stages of embryonic development. Kidney agenesis or malformations are the most commonly associated with unilateral kidney agenesis. Ovaries may be ectopic in 16–19% of MRKHS patients. Primary amenorrhoea, due to the absence of the uterus, is the most common presentation. Female karyotype confirmation is mandatory to differentiate it from complete androgen insensitivity syndrome and 17-alpha-hydroxylase deficiency. The management of MRKHS is multidisciplinary in order to encompass psychological, medical and surgical issues.

**Case presentation:**

A four-year-old girl, presented to the emergency department complaining of left groin swelling noted 2 days earlier. The patient had recently been evaluated for an episode of acute abdominal pain and vomiting, with a final diagnosis of right ovarian torsion. At that time, the ultrasound imaging was not able to identify the left kidney, the left ovary and uterus. Surgical abdominal exploration confirmed the right ovarian torsion and was not able to identify the left kidney and the left ovary. Only a remnant of the uterus was present. Therefore, the right ovary was removed, and a diagnosis of MRKHS was made. Ultrasound imaging showed a left inguinal hernia. The hernial sac consisted of a solid oval vascularized formation suggestive of an annexe. The patient underwent a surgical procedure to correct the left inguinal hernia. In the operating setting, the presence of a vascularized, ectopic ovary carrying the tuba inside the hernial sac was observed.

**Conclusions:**

In front of a patient with ovarian torsion and anatomical features suggestive of MRKHS, both the ovaries should always be searched for, with a high suspicion threshold for extrapelvic ovary. Identifying the ectopic ovary, in this case, helped to preserve patient fertility, avoiding a possible torsion.

## Learning points

1. Mayer-Rokitansky-Küster-Hauser Syndrome (MRKHS) originates from abnormal embryonic development of the müllerian structures, resulting in congenital hypoplasia/aplasia of the uterus, the cervix and the upper 2/3 of the vagina, in females with normal ovaries and fallopian tubes.
2. MRKHS is the second most common cause of primary amenorrhoea after the constitutional pubertal delay in young females with normal secondary sexual characteristics and 46 XX karyotype.
3. In MRKHS, both the ovaries are always present and should be actively investigated. They may be ectopic and more prone to torsion. A missed visualization at ultrasound imaging or even at surgical exploration suggests an extrapelvic location.

## Background

Mayer-Rokitansky-Küster-Hauser syndrome (MRKHS) refers to congenital hypoplasia/aplasia of the uterus, the cervix and the upper 2/3 of the vagina, in females with normal ovaries and fallopian tubes, secondary sexual characteristics and 46 XX karyotype [1].

It is reported to have an incidence of 1:4500 women [2].

This condition (also known as müllerian agenesis), originates from abnormal development of Müller’s paramesonephric ducts in the early stages of embryonic development. It can be sporadic, but the recurrence of familial cases supports the autosomal dominant inheritance theory, with variable penetrance. As the genetics of MRKHS remains elusive, oligogenic or polygenic inheritance has also been hypothesised [3].

MRKHS is commonly classified in two types based on associated anatomical features: isolated (type 1, about 44% of cases) or linked with other anatomical malformations (type 2, about 56% of cases). Kidney agenesis or malformations are the most commonly associated (50% of patients) with unilateral kidney agenesis reported in 15% of all MRKHS. Vertebral malformations are also common. Auditory, cardiac and extremity anomalies are possible but less frequent [1, 4]. Ovaries may be ectopic in 16–19% of MRKHS patients.

Primary amenorrhoea, due to the absence of the uterus, is the most common presentation.

Nevertheless, MRKHS can be suspected in younger females, in the presence of urinary malformations associated with a variable-grade aplasia/hypoplasia of müllerian structures. Female karyotype confirmation is mandatory to differentiate it from complete androgen insensitivity syndrome and 17-alpha-hydroxylase deficiency.

Functional ovarian anomalies as a polycystic ovarian syndrome, advanced puberty, androgenic excess and also rarely ovarian cancer have been described [5, 6].

Ovarian inguinal hernias, which occasionally occur in females [7], are not uncommon in young patients with MRKHS. Ectopic ovarian torsion and infarction are reported in 2–33% of MRKHS patients presenting with non-reducible groin swellings, and may result in a sunk ovary; salpingitis can also be observed [8].

The management of MRKHS is multidisciplinary in order to encompass psychological, medical and surgical issues. Surgery has to be considered in patients sexually active who prefer surgical creation of a vaginal canal but can also be necessary earlier in order to prevent and relieve ovarian torsion.

## Case presentation

A four-year-old girl presented to the emergency department complaining of left groin swelling noted 2 days earlier.

No pain, fever, vomiting, dysuria or diarrhoea were reported.

The patient had recently been evaluated for an episode of acute abdominal pain and vomiting, with a final diagnosis of right ovarian torsion. At that time, the ultrasound imaging was not able to identify the left kidney, the left ovary and uterus. Surgical abdominal exploration confirmed the right ovarian torsion and was not able to identify the left kidney and the left ovary. Only a remnant of the uterus was present.

Therefore, the right ovary was removed, and a diagnosis of Mayer-Rokitansky-Kuster-Hauser Syndrome (MRKHS) Type 2 was suspected. Karyotype resulted in female, 46 XX.

**Fig. 1:**
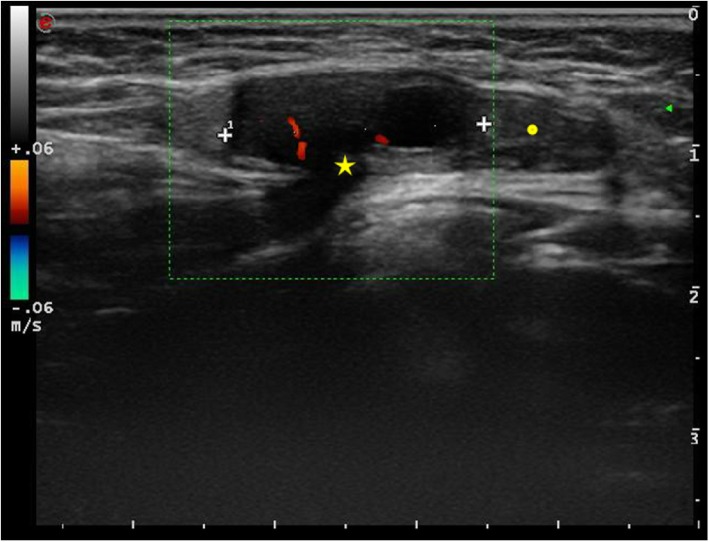
The ultrasound imaging shows a 3 cm hernial sac bulging through a 5 mm breach (star) from the inguinal canal. The hernial pouch consisted of adipose tissue in the cranial portion (point), followed by a solid elliptical formation that appears vascularized at color doppler

**Fig. 2:**
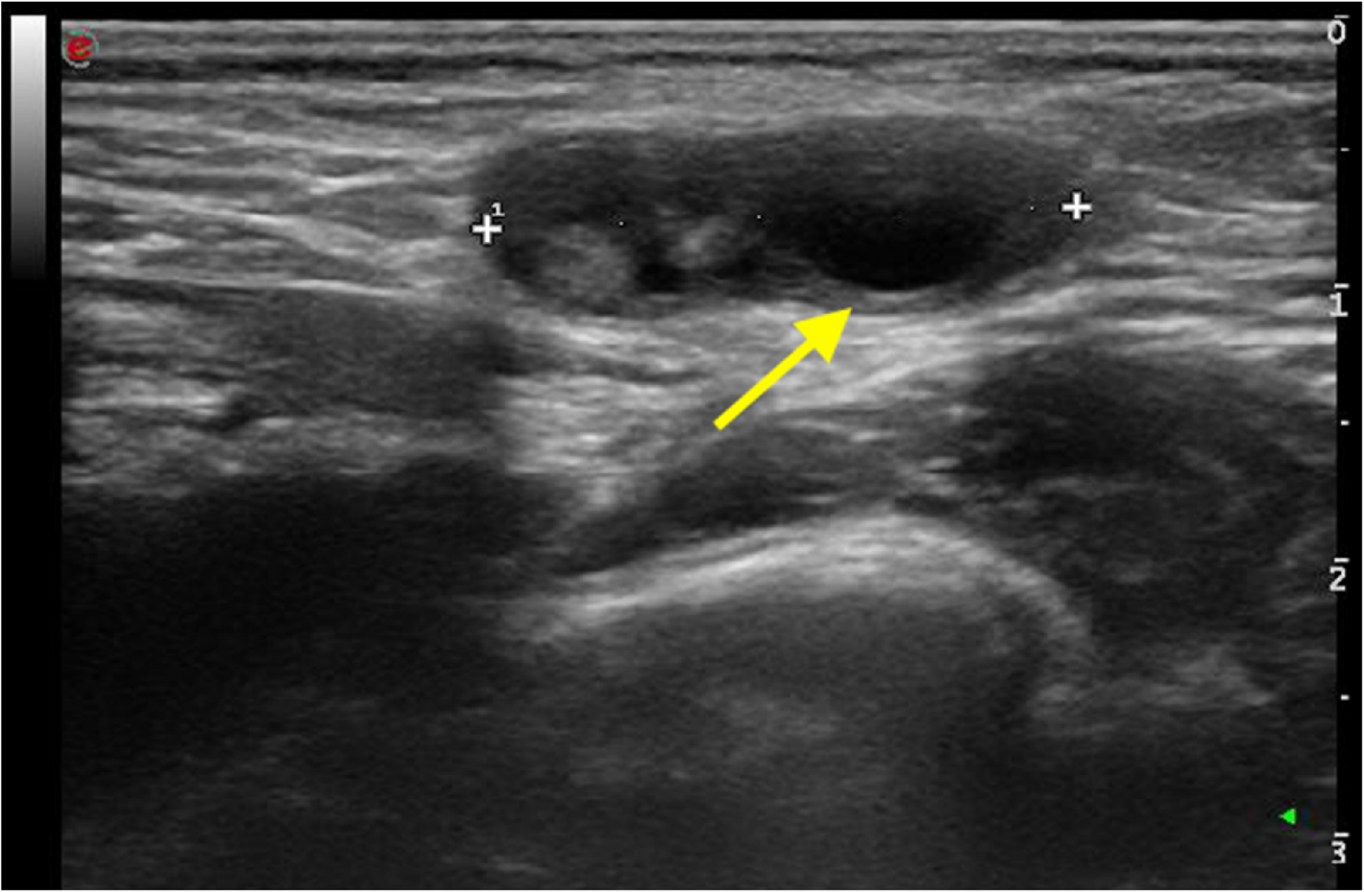
The observation of the follicles within the solid formation (arrow), as well as its tense-elastic texture and the presence of vascularization at Color Doppler, made likely the hypothesis of an annexe

At physical examination, the swelling in the left groin was confirmed. A little palpable mass, mimicking a prepubertal testis retained in the inguinal canal, was noted. At palpation, no pain was elicited. Ultrasound imaging showed a left inguinal hernia with a 5 mm breach (Fig. 1). The hernial sac consisted of adipose tissue in the cranial portion, followed by a solid oval vascularized formation suggestive of an annexe (Fig. 2). A surgical consultation was requested, and the patient underwent a surgical procedure to correct the left inguinal hernia. In the operating setting, the presence of a vascularized, ectopic ovary carrying the tuba inside the hernial sac was observed; both the structures were reduced into the abdominal cavity. There were no surgical complications, and in post-operative, the patient had a rapid and complete recovery.

CEA, CA 19–9, CA 125, Alpha-fetoprotein and Beta HCG dosages resulted in normal ranges.

## Discussion and conclusion

MRKH Syndrome originates from a failed fusion of the müllerian ducts that causes a lack of their derivatives. Given the different embryological origins, the ovaries are not involved. In front of a patient with ovarian torsion and anatomical features suggestive of MRKHS, both the ovaries should always be searched for, with a high suspicion threshold for extrapelvic ovary. Surgical exploration may not identify an ectopic ovary. Ectopic ovary torsion is likely in these patients and should always be actively investigated. In particular, in our case, MRKHS Type 2 diagnosis was correct given the absence of one kidney and the uterus, but the missed finding of the left ovary during abdominal surgical exploration lead to the wrong hypothesis of associated ovarian agenesis. Identifying the ectopic ovary, in this case, helped to preserve patient fertility, avoiding a possible torsion. In conclusion, ovarian agenesis does not pertain to MRKHS clinical picture. It is necessary to look for an ectopic ovary in all MRKHS patients. Surgical exploration may not identify an ectopic ovary; therefore, a high suspicion threshold for extrapelvic ectopic ovary and proper surgical management are required to avoid torsion and preserve fertility in these patients. Primary amenorrhoea, due to the absence of the uterus, in pubertal females with normal thelarche and adrenarche, is the most common presentation. Hormonal therapy is inappropriate, given the presence of functioning ovaries. Testosterone and other androgens levels, as well as estrogens and gonadotropins, are normal. The most challenging aspect for these patients is infertility. In vitro fertilization is possible using the patients’ oocytes with a gestational surrogate; cases of children born to mothers with MRKHS after uterus transplantation are also described.

## Data Availability

All data generated or analysed during this study are included in this published article.

## Abbreviation

MRKHS: Mayer-Rokitansky-Küster-Hauser syndrome
